# Treatment of Unstable Tibia Shaft Fractures in Children With Elastic Stable Intramedullary Nails or Minimally Invasive Plate Osteosynthesis: Comparative Study on 2 Treatment Options

**DOI:** 10.1097/BPO.0000000000003077

**Published:** 2025-08-20

**Authors:** Michał Maciejewski, Jan Klincewicz, Piotr Janusz

**Affiliations:** * Department of Pediatric Orthopedics and Traumatology, Mother and Child Care Center; † Department of Spine Disorders and Pediatric Orthopaedics, Poznan University of Medical Sciences, Poznan, Poland

**Keywords:** tibia fracture in children, elastic-stable intramedullary nail, minimally invasive plate osteosynthesis

## Abstract

**Introduction:**

Tibial shaft fractures are common fractures in children. The optimal management should be selected based on the fracture type, the child’s weight, and the presence of open growth plates. When surgical treatment is indicated, elastic-stable intramedullary nailing (ESIN) is considered the generally preferred method in children with open physes. However, in some cases, alternative treatment may be required.

**Aim:**

The aim of this study was to compare the outcomes of tibial shaft fractures in children treated with ESIN versus minimally invasive plate osteosynthesis (MIPO).

**Methods:**

Fifty-nine children were treated for unstable tibial shaft fractures between 2018 and 2023 (27 with MIPO and 32 with ESIN). Patients’ demographics, fracture type, surgery duration, and complications were recorded based on medical records. Bone healing, tibial axis, and implant position were assessed on follow-up radiographs. Functional outcomes were evaluated using the EFAS Questionnaire at 3.7±1.8 years (range: 1.0 to 6.8) after surgery.

**Results:**

Bone union was achieved in all patients. The total complication rate was 7.4% in the MIPO group and 15.6% in the ESIN group (*P*=0.4365). No reoperations were required in the MIPO group, while 12.5% of patients in the ESIN group required reoperation (*P*=0.1176). A plaster cast was applied in 46.9% of ESIN patients and in none of the MIPO patients. The surgery duration was significantly longer in the MIPO group (79.0 vs. 41.8 min in the ESIN group; *P*=0.0001). There was no significant difference in the final tibial axis at the final follow-up (2 ESIN patients underwent reoperation for axis correction). There was no significant difference in EFAS Questionnaire scores: 38.1±2.1 in the MIPO group versus 36.5±3.9 in the ESIN group (*P*=0.1235).

**Conclusions:**

Minimally invasive plate osteosynthesis is a promising alternative to elastic-stable intramedullary nails in the most severe, unstable tibial shaft fractures. Plating provides better stabilization without the need for a plaster cast; however, surgery time is longer.

**Level of Evidence:**

Level III.

Tibial shaft fractures account for ∼15% of all fractures in children, making them one of the most common long-bone fractures in the pediatric population.^1^ It is the third in frequency among long bone fracture in children, following fractures of the forearm and humerus.^2^ The mechanism of injury leading to tibial shaft fractures in children varies significantly depending on age and the energy of trauma, resulting in different fracture types.^1,3^ Depending on the type of fracture, its biomechanical characteristics, associated with it stability, soft tissue injuries, accompanying fibular fractures, and other factors such as the child’s weight, age, and presence of active growth plates the most appropriate management strategy should be selected to optimize healing and functional recovery.^3^

There are various treatment options, ranging from conservative management to surgical procedures such as the use of elastic-stable intramedullary nails (ESIN) also referred to as flexible intramedullary nails (FIN), external fixators, locking plates, and ridge intramedullary nails in children with closed growth plates.^4^ Currently, ESIN is considered the generally preferred method for treating tibial fractures in children with open growth plates. This method provides high rates of bone union without damaging the growth plate, allows early return to physical activity, has low infection rates, and results in minimal scarring. However, in certain cases—such as oblique spiral or comminuted fractures located near the physis, or in children weighing more than 50 kg—ESIN fixation may be insufficient. In such situations, additional stabilization with a plaster cast or alternative procedures such as plate osteosynthesis may be required.^4,5^ Recently, minimally invasive plate osteosynthesis (MIPO) has gained increasing attention as an alternative to open plate osteosynthesis.^6–8^

## AIM

The aim of the study was to compare the outcomes of the tibial shaft fractures in children treated with ESIN versus MIPO.

## METHODS

Fifty-nine patients who underwent operative treatment for unstable tibial shaft fractures between January 2018 and November 2023 at a single pediatric trauma center were included in the study. There were 38 boys and 21 girls, with a mean age of 12.7 years (range: 6.2 to 17.9). All fractures were oblique or comminuted, and 88.1% were associated with an additional fibular fracture.

Twenty-seven patients were treated with minimally invasive plate osteosynthesis (MIPO), and 32 with elastic-stable intramedullary nailing (ESIN). The choice of treatment was based on the surgeon’s preference. A plaster cast was applied when intraoperative stabilization was deemed insufficient.

After Institutional Review Board approval (No. 818/21), a retrospective review of medical records was conducted. Patients were routinely assessed during hospitalization, at follow-up visits, and during the second planned hospitalization for implant removal. Each time radiologic and clinical examination was performed. Patient demographics, type of injury, fracture classification,^9^ surgical method, duration of surgery, implant removal time, and complications were collected from medical records. On the basis of the medical history, clinical findings, and operative report, there was no evidence to suggest that any of the fractures were open fractures. Clinical examination included wound healing evaluation, range of motion assessment, and measurement of lower limb length discrepancy (LLD) in a standing position. Bone healing, tibial axis, and implant position were evaluated using radiographs obtained during follow-up. Delayed union was defined as the absence of bone union after 3 months, and nonunion as the absence of union after 6 months. The tibial axis (TA) in the coronal plane was assessed as the mechanical tibial coronal axis. The sagittal axis was measured using the anterior distal tibial angle (ADTA). Comparison of the TA and ADTA between both groups was performed based on the radiograph taken during implant removal. Tibial axis deviations requiring surgical correction were defined as >10 degrees at any radiograph taken during follow-up. For the comparison of radiologic tibial axis measurements between groups, measurements based on radiographs taken at the time of implant removal were used. Severe range-of-motion limitation was defined as >20 degrees compared with the contralateral side. A lower limb length difference >1 cm, measured at the time of implant removal, was considered significant. High-energy trauma was defined as a fall from a height greater than the patient’s own height. An unstable fracture was defined as a comminuted or spiral fracture, or a fracture that could not be properly reduced in the Emergency Department, or one in which loss of reduction occurred during follow-up.

All included patients were contacted by phone or e-mail, and the functional status of the operated limb was assessed using the Polish version of the EFAS Questionnaire. All patients or their family members (59/59) completed the questionnaire. Functional evaluation was performed at a mean of 3.7±1.8 years (range: 1.0 to 6.8) after surgery.

### Surgical Technique

**FIGURE 1:**
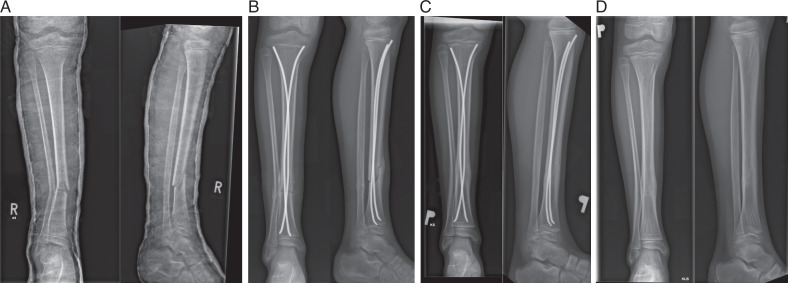
Fractures treated with ESIN: (A) before surgery, (B) after surgery, (C) 2 months after surgery, and (D) after implant removal.

The ESIN group was treated using the standard technique through anterolateral and anteromedial surgical approaches to the proximal tibial metaphysis. After opening the medullary canal with a trocar, 2 prebent elastic titanium intramedullary nails were inserted to the level of the fracture. Closed reduction was performed under fluoroscopic guidance, followed by advancement of the nails into the distal tibial metaphysis. Example radiographs of patients treated with ESIN are presented in Figure 1.

**FIGURE 2:**
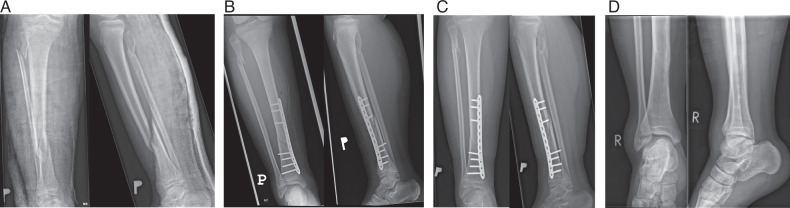
Fractures treated with MIPO: (A) before surgery, (B) after surgery, (C) 2 months after surgery, and (D) after implant removal.

The MIPO group was treated using the technique described in the AO Surgery Reference for Pediatric Trauma.^10^ The first incision was made on the anteromedial side of the distal third of the tibia. After incising the skin and carefully protecting the neurovascular bundle, the plate was inserted using a single locking screw drill sleeve. Fracture reduction was achieved under fluoroscopic guidance, and additional small incisions were made over the plate holes for locking screw insertion. If the reduction was unsatisfactory or unstable, lag screws were inserted either through the plate or independently. Lag screws were placed before the locking screws. If a displaced fibular fracture was present, the initial incision was made on the lateral side at the level of the fracture. The fracture site was exposed, reduced, and stabilized using screws or a plate. Example radiographs of patients treated with MIPO are presented in Figure 2.

### Statistical Analysis

Mean values and SDs were calculated. The Kolmogorov-Smirnov test was used to assess for normal distribution. The *t* test was used to compare normal distribution data and the Mann-Whitney test was used to compare non-normal distribution data. The χ^2^ test was used for nominal data. Results were considered significant at *P*<0.05. Statistical analyses were performed using MedCalc version 12.7.8.0.

## RESULTS

The groups were comparable in terms of age, sex, affected side, trauma energy, and follow-up duration. Patients treated with MIPO were significantly taller and heavier than those treated with ESIN. There were no differences in the incidence of concomitant fibular fractures or in AO fracture classification between the groups, Table 1.

**TABLE 1:** Comparison of the Evaluated Groups

	MIPO N=27	ESIN N=32	*P*
Age at the surgery (y)	13.1±1.8	12.0±2.9	0.0781*
	9.8-16.2	6.2-17.9	
Weight (kg)	69.5±15.2	57.8±21.2	**0.0149** *
	42.5-108	27-117	
Height (cm)	169.5±11.1	160.4±16.2	**0.0160** †
	147-186	130-190	
Sex: M/F	21/6	17/15	0.0896‡
Affected side R/L	16/11	16/16	0.6535‡
High energy trauma y/n	22/5	23/9	0.5411‡
Tibia and fibula fracture/tibia fracture	25/2	27/5	0.5698‡
AO classification 42t-D/5.1 **/** 42t-D/5.2	19/8	28/4	0.1923‡
Follow-up with EFAS functional questionnaire (y)	3.7±1.9	3.6±1.6	0.9317*
	1.0-6.8	1.3-6.7	

* Mann-Whitney test,

† *t* test,

‡ χ^2^ test, bolded: *P*<0.05

All patients achieved bone union. In 2 patients treated with ESIN, bone union was delayed. The overall complication rate was 7.4% in the MIPO group and 15.6% in the ESIN group (*P*=0.4365). No reoperations were required in patients treated with MIPO, while 12.5% of patients in the ESIN group underwent reoperation, *P*=0.1176. All complications are summarized in Table 2.

**TABLE 2:** Treatment Complications in the Evaluated Groups

	MIPO N=27	TEN N=32
Total complication number	2	5
Superficial wound infection	1	
Fibula axis deviation	1	
Implant migration		1*
Skin laceration with the implant		1*
Axis deviation >10 degree		2*
Range of motion limitation >20vdegree		1
Reoperations number	0	4

* Unplanned reoperation.

The plaster cast was applied in 46.9% patients treated with ESIN and in none of the patients treated with MIPO, Table 3. Time of the surgery was significantly longer in patients treated with MIPO 79.0 versus 41.8 minutes in patients treated with ESIN, *P*=0.0001. Implant removal occurred at a mean of 11.0 months in MIPO patients and 9.1 months in ESIN patients, *P*=0.0137. There was no difference in final tibia axis in both the sagittal and the coronal plane (2 patients from the ESIN group were reoperated before final evaluation due to axis deviation), Table 3. No patient from any of the studied groups demonstrated a lower limb length discrepancy >1 cm. The clinical and radiologic outcomes were assessed during the hospitalization for implant removal.

**TABLE 3:** Treatment Differences Between the Evaluated Groups

	MIPO N=27	ESIN N=32	*P*
Time of surgery (min)	79.0±21.1	41.8±16.2	**0.0001** *
	40–113	19–86	
Implant removal time (mo)	11.0±3.7	9.1±6.1	0.0137*
	6–22	4–33	
Plaster cast	0/27	15/32	**0.0001** †
Axis deviation at implant removal, coronal plane (deg.)	1.7±1.0	1.6±1.1	0.2846*
	0–4	0–5	
ADTA at implant removal, sagittal plane (deg.)	80.7±1.3	80.7±1.4	0.7215*
	78–82	77–82	
Fibula fixation	21	2	**0.0001** †

* Mann-Whitney test,

† χ^2^ test, bolded: *P*<0.05, ADTA.

There was no significant difference in EFAS Questionnaire scores: 38.1±2.1 in patients treated with MIPO and 36.5±3.9 in those treated with ESIN (*P*=0.1235). The EFAS Questionnaire was obtained 3.7±1.9 years after surgery in the MIPO group and 3.6±1.6 years in the ESIN group, *P*=0.9317.

## DISCUSSION

Our study does not seek to challenge the well-established, evidence-based standards for the management of pediatric tibial fractures. Conservative management remains the primary approach and if surgical treatment is necessary, the treatment of choice for children with open growth plates is the use of ESIN.^4,11^ ESIN works by converting shear forces into compressive forces, yielding excellent outcomes in transverse fractures.^11^ However, in some cases, maintaining proper length and preventing angular deformities can be difficult with this method alone. This is particularly important in cases of highly oblique, spiral, or comminuted fractures, nearer to the metaphysis, especially in children with a high body mass. Typical alternatives to ESIN, such as additional casting, open plate osteosynthesis, or external fixation, are associated with specific complications and inconveniences for the patient.^3,4^

There are several studies concerning the application of the MIPO technique in pediatric tibia fractures.^6–8^ This method is one of the possible treatment options for pediatric tibial fractures, as referenced by the AO Foundation.^10^ The main advantage over open plating is limited soft tissue dissection while still providing adequate fracture stabilization,^6^ making it more similar to ESIN in terms of tissue damage and healing disruption, but providing better stability.

Yusof and colleagues presented the first case series of the application of MIPO in tibial fractures in 16 children. They reported one case of a superficial infection, one case of discomfort over the plate, and one case of a 15 mm leg length discrepancy.^7^ Masquijo^8^ described 11 tibial fractures in children treated with MIPO, reporting only superficial infection and no cases of nonunion or reoperation. Ozkul et al^12^ reported a series of 14 pediatric patients treated with MIPO for open tibia fractures, with 2 cases of superficial infection and 2 cases of delayed union. The largest study presented by Murphy and colleagues, who reported 28 tibial fractures (10 open fractures) in 26 skeletally immature patients. They described 2 superficial infections, one 15 mm leg length discrepancy, and one case of fasciotomy at the time of plate fixation due to trauma-related compartment syndrome.^6^

In our MIPO group, we observed a 3.7% rate of superficial infection, which is lower than the 7% to 14% reported in previous studies. We also did not find delayed union, which has been reported in earlier publications.^6,8,12^ According to our criteria, Ozkul et al^12^ reported 10 cases, and Yousef et al reported 4.^6,8^ However, in our study, there were no patients with open fractures, and not all fractures were caused by high-energy trauma. High-energy trauma was the predominant type of injury 81.5%. Nevertheless, the primary reason for choosing MIPO stabilization was the biomechanics of the fracture and the lack of stability after reduction.

In the MIPO method, both medial^6,8^ and lateral^12^ plate placements are described. There is a hypothesis that lateral plate placement, due to the presence of muscle tissue, may be more beneficial for healing and may reduce the risk of infection.^12^ However, this issue has not been fully proven.^6,12^ Nevertheless, we preferred to use the medial approach to stabilize the tibia, based on AO Foundation recommendations.

We found a relatively high complication rate in the ESIN group in our study. Although the complication rate for ESIN varies significantly across studies, a meta-analysis conducted by Stenroos et al^13^ reported a similar overall complication rate of 24%, with a higher prevalence of delayed unions and fewer reoperations compared with our findings. Similarly, Fanelli et al^14^ describe a total complication rate of 28%.

Another issue is the plaster cast used after the ESIN stabilization. This remains a controversial topic. Some authors always recommend casting for a few weeks after surgery,^11^ while others do not apply any immobilization,^15^ and others use it in selected cases.^16^ Nevertheless, it is still widely used, especially in heavier children with unstable fractures.

No additional immobilization in patient treated with MIPO, regardless of the child’s weight. Others reports on MIPO also indicate that no immobilization was used.^6,7^

To our knowledge, this is the first study comparing ESIN with MIPO for the treatment of unstable tibial fractures in children. Both methods are minimally invasive and spare the growth plate, thus may be used for growing children. Although the MIPO group tended to include older, heavier and taller, other characteristics were well matched, allowing for valid comparisons between the groups.

The MIPO technique appears to be associated with fewer issues related to implant migration and a reduced number of required reoperations. None of these trends reached statistical significance in our group. There was one case of superficial infection in the MIPO group, which resolved with oral antibiotics and did not require surgical intervention. This finding is consistent with other studies on MIPO.^6–8^

The duration of ESIN surgery in our study was comparable to that reported in other studies.^16^ However, the surgery time was nearly twice as long in patients treated with MIPO. In contrast, the ability to provide more stable fracture immobilization, offering better rotational stability, may help offset this disadvantage. Another factor that may have contributed to the prolonged surgery time is that some of our patients also underwent fibular fracture stabilization. The necessity of this procedure remains controversial. Although it may aid in restoring proper length and rotation at the injury site, it also increases surgical trauma. However, clear indications for this approach in pediatric patients have not yet been definitively established.

The functional outcomes measured with the EFAS questionnaire were comparable between the groups. To our knowledge, this is the first study evaluating the functional outcomes of MIPO treatment for tibial fractures in children using a validated scale.

One of the limitations of this study is the relatively small study group. However, to our knowledge, there is no other study that includes a larger number of pediatric patients treated with MIPO.^6–8^ Another limitation is the absence of a standardized treatment protocol, with decisions based solely on surgeon preference. In the case of ESIN, there was also no protocol for the application of a plaster cast. Although this reflects real-life clinical practice, it may have influenced the outcomes. An additional limitation is that lower limb length discrepancy (LLD) was assessed only clinically. Although we did not detect any cases of LLD, our follow-up may have been insufficient to identify growth abnormalities, as most patients were not monitored until skeletal maturity, and no standardized long-film radiologic evaluation of both lower limbs was performed. However, this limitation was also present in other studies concerning MIPO.^8^

Despite these limitations, our study provides valuable insights into the comparison of ESIN and MIPO. MIPO is an alternative treatment for unstable tibia fractures in children, particularly in cases requiring open plate osteosynthesis. This technique involves minimal soft tissue dissection and does not disrupt the fracture healing process. The lack of need for plaster cast application enables early joint mobility and helps maintain muscle strength. This is especially important in obese patients with tibial fractures, as they tend to sustain more severe injuries, which may predispose them to greater morbidity and complications^17^

## CONCLUSIONS

Minimally invasive plate osteosynthesis is a promising alternative to elastic-stable intramedullary nails in the most severe, unstable tibial shaft fractures. Plating provides better stabilization without the need for a plaster cast; however, surgery time is longer.
